# Interlimb transfer and generalisation of learning in the context of persistent failure to accomplish a visuomotor task

**DOI:** 10.1007/s00221-019-05484-4

**Published:** 2019-02-13

**Authors:** D. A. E. Bolton, A. R. Buick, T. J. Carroll, R. G. Carson

**Affiliations:** 1 Department of Kinesiology and Health Sciences, Utah State University, Logan, USA; 2 School of Psychology, Queen’s University Belfast, Belfast, UK; 3 School of Human Movement and Nutrition Sciences, The University of Queensland, Brisbane, Australia; 4 Trinity College Institute of Neuroscience, School of Psychology, Trinity College Dublin, Dublin 2, Ireland

**Keywords:** Cross education, Bilateral transfer, Motor control, Motor learning, Movement disorder

## Abstract

Transfer, in which capability acquired in one situation influences performance in another is considered, along with retention, as demonstrative of effectual learning. In this regard, interlimb transfer of functional capacity has commanded particular attention as a means of gauging the generalisation of acquired capability. Both theoretical treatments and prior empirical studies suggest that the successful accomplishment of a physical training regime is required to bring about generalised changes that extend to the untrained limb. In the present study, we pose the following question: Does interlimb transfer occur if and only if the training movements are executed? We report findings from JG—an individual recruited to a larger scale trial, who presented with (unilateral) deficits of motor control. We examined whether changes in the performance of the untrained right limb arose following practice undertaken by the impaired left limb, wherein the majority of JG’s attempts to execute the training task were unsuccessful. Comparison was made with a group of “control” participants drawn from the main trial, who did not practice the task. For JG, substantial gains in the performance of the untrained limb (registered 3 days, 10 days and 1 year following training) indicated that effective learning had occurred. Learning was, however, expressed principally when the unimpaired (i.e. untrained) limb was utilised to perform the task. When the impaired limb was used, marked deficiencies in movement execution remained prominent throughout.

## Introduction

It is a widespread, if generally implicit, assumption that motor learning is contingent upon the engagement of specific motor control processes (e.g. Sainburg et al. 2016). Although Willingham (1998) first articulated the distinct hypothesis that motor learning emerges directly from the basic architecture of motor control, the premise had long been fundamental to traditional theories of skill acquisition (Newell 1991). There are a variety of ways in which the effectiveness of skill acquisition can be determined. Transfer, in which capability acquired in one situation influences performance in another is considered, along with retention, as demonstrative of effectual learning (e.g. Magill 2004; Soderstrom and Bjork 2015). In this regard, interlimb transfer of functional capacity has commanded particular attention as a means of gauging the generalisation of acquired capability (Ruddy and Carson 2013).

In Willingham’s original (1998) scheme, “learning occurs if and only if a movement is executed” (page 565). More specifically, the learning that occurs is based upon the action that is actually generated. At first glance, evidence that “mental practice” enhances the quality of physical performance (e.g. Pascual-Leone et al. 1995) would appear contrary to this assertion. That which is conspicuous, however, is that with respect to interlimb transfer, the beneficial effects of mental practice are typically restricted to tasks that require that a sequence of spatially discriminable movements be learned (e.g. Land et al. 2016), or include elements that are amenable to symbolic representation (e.g. Lohse et al. 2010). A notable exception is the observation by Yue and Cole (1992) that imagined unilateral isometric contractions (abduction of the fifth digit) brought about bilateral increases in maximal abduction force. This specific instance notwithstanding, the overall pattern of outcomes is consistent with the more general finding that the degree to which a task involves cognitive components is a significant predictor of the gains in performance that can be realised through mental practice (Driskell et al. 1994; see also; Malfait and Ostry 2004). Correspondingly, there is little evidence to suggest that mental practice engenders the interlimb transfer of effector (e.g. joint or segment direction) specific capability—as it is expressed in terms of movement kinetics or kinematics. In a similar vein, following observation of a spatial–temporal pattern of elbow flexions and extensions, gains in performance realised by the non-engaged limb (i.e. of the observer) are greater when the patterns of movement are defined in extrinsic coordinates (visual and spatial location of the target waveform), than when defined in term of the required pattern of muscle activity (sequence of activation of elbow flexors and extensors). In contrast, following physical practice, the performance of the non-engaged limb is superior when the equivalent pattern of muscle of activity is produced—rather than the equivalent visuospatial pattern (Gruetzmacher et al. 2011; see also; Hayes et al. 2012). With respect to interlimb transfer therefore, there is relatively little available evidence to contradict the hypothesis that physical practice is required to bring about generalised changes in effector (e.g. joint or segment direction) specific capability that extend to the untrained limb.

Following Willingham (1998), one may pose a related question: Does interlimb transfer occur if and only if the training movements are executed? The present case report concerns a unique opportunity to address this question that arose in the context of a larger scale trial. The report concerns an individual who exhibited conspicuous abnormalities in the control of his left arm. These included an impaired ability to produce wrist extension torques—observed in the context of a task that required combinations of torques in wrist flexion–extension and radial–ulnar deviation be produced to acquire visual targets. Our present focus is upon gains in the performance of the untrained right limb that arose following practice undertaken by the impaired left limb, wherein many of the attempts to execute the training task were unsuccessful.

## Materials and methods

JG is a right-handed (Oldfield 1971) man enlisted originally at age 69 years, as part of a larger study that comprised approximately 100 young (18–30 years) and 100 older (over 65 years) people. All prospective participants first answered questions by telephone and via screening documents (mailed to their homes), to identify and exclude persons who had been diagnosed by a physician with a neurological disorder (such as Stroke, Parkinson’s disease, Alzheimer’s disease and Spinal cord injury). As JG indicated that no such diagnosis had been made (notwithstanding regular visits to his physician), he was enrolled in the study. Upon arrival he presented with a number of clear motor abnormalities. A slow shuffling gait was apparent along with some freezing of speech. In terms of upper arm function, JG demonstrated hypertonia (rigidity) particularly on his left side and exhibited difficulty removing his jacket. During our intake interview, JG was again asked if he had been diagnosed with any form of neurological disorder, to which he answered ‘No’. This remained his response when he was asked 1 year later. He did, however, mention that he suffered a fall onto his left side several years earlier but with no reported fractures or lingering neuromuscular consequences. When attempting to isolate specific wrist muscles for electromyography (EMG) electrode placement, resistance to passive movement of the left arm was observed to be greater than that for the right arm. JG exhibited difficulty generating isolated extension of the wrist (instead he tended to abduct his arm). JG exhibited profuse sweating during training and assessment.

During our initial intake session, we administered the Montreal Cognitive Assessment (MOCA) (Nasreddine et al. 2005) as a standard screening tool, on which JG received a score of 23/30. This score places him within a range that has in some cases been classified as mild cognitive impairment (Coen et al. 2011; Luis et al. 2009). The most prominent deficit was with respect to delayed recall for un-cued words (score: 1/5). JG responded appropriately to questions and appeared engaged throughout the entire study. Moreover, in all testing and training sessions, JG demonstrated high levels of motivation.

JG and participants in a control group (see below) provided written informed consent to the procedures, which were approved by the relevant Queen’s University Belfast Ethics Committee and conducted in accordance with the Declaration of Helsinki. We take this opportunity to note that JG did not wish for representations to be made on his behalf, which may have led to specialist medical consultations or brain imaging.

### Experimental setup

**Fig. 1:**
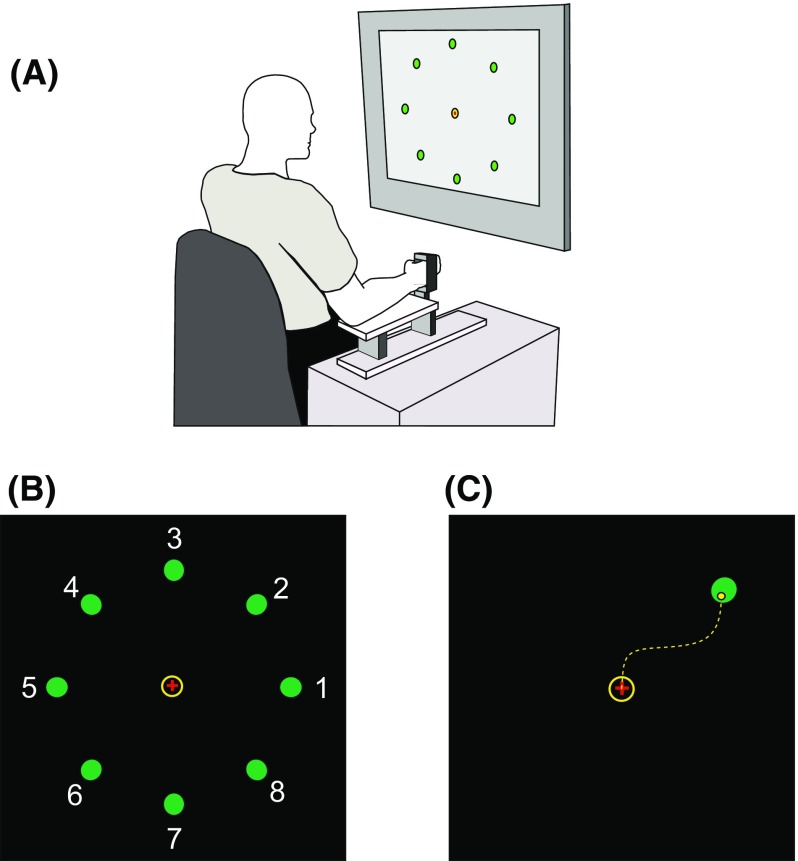
**a** Apparatus. Each hand was securely fixed within a manipulandum that was instrumented to transduce torques generated in two degrees of freedom at the wrist. These controlled a visual cursor displayed on a screen such that via flexion–extension torques produced left–right motion, and radial–ulnar deviation torques produced up–down motion. **b** Protocol. It was necessary that the cursor remain in the home zone (yellow circle) for a continuous period of 200 ms in order for a trial to commence. One of eight visual targets (represented as green dots) equally spaced around the start position was presented on each trial. **c** Acquisition. Following presentation of a target, the participant moved the cursor to the target as quickly as possible. If the cursor was then maintained continuously within the target radius for 50 ms, a tone signalled successful target acquisition

The participant was seated with his upper limbs supported on horizontal platforms, with lateral motion prevented by foam-covered posts placed on either side of each arm. The head, neck, torso and feet were also fully supported. The forearms were stablised in mid-pronation and the elbows semi-flexed (100–120°). Each hand was securely fixed within a manipulandum (see Fig. 1a) that was instrumented to transduce isometric torques generated in two degrees of freedom (i.e. flexion–extension, and radial–ulnar deviation) at the wrist. The outputs from the transducers were low-pass (analog) filtered at 8 Hz, and then sampled at 2000 Hz. A computer monitor was positioned at 1 m in front of the participant, with the centre of the screen at eye level. All stimulus presentation and data collection procedures were implemented using custom Labview (National Instruments, Austin, TX) routines.

## Experimental procedure

Maximal voluntary torque (MVT) data were first collected separately for each limb, and for each direction of applied torque (i.e. flexion; extension; radial deviation; ulnar deviation). To establish MVT, the participant was instructed to apply as much “force” as possible in the specified direction for approximately 3 s. Three attempts were made for each direction, with successive attempts separated by a rest period of 30 s. The peak torque achieved during each trial was calculated, and these values averaged over the three trials to provide a measure of MVT (i.e. separately for each limb and direction). These MVTs were used subsequently to scale the magnitude of the torques required to acquire visual targets in the behavioural tasks.

The participant was instructed to use torques generated at his wrist to move a circular yellow cursor on the computer monitor (Fig. 1b). The cursor responded to the joint torques in an intuitive way such that extension torques generated by the right limb, and flexion torques generated by the left limb, would move the cursor to the right. For both limbs, radial deviation torques moved the cursor upwards, and so on. The participant was asked to move the cursor to acquire a circular green target that appeared in pseudo-random order at one of eight different locations (distributed at 45**°** intervals around the circumference of a circle) surrounding a central ‘home’ zone (Fig. 1b). This home zone represented the torque levels registered when the limb was at rest. For targets 2, 4, 6, and 8, the torque necessary to displace the cursor to acquire the target was defined in relation to the MVT values obtained in two directions. For example, when using the left limb to acquire target 2, the required torque was determined (i.e. multiplied by the cosine of 45**°**) with respect to both the MVT for radial deviation, and the MVT for flexion (or relative to the MVT for extension when the right limb was used). The diameter of the cursor and the target each corresponded to approximately 1.15° of visual angle (a little more than double the retinal image size for the moon).

There are certain practical advantages that accrue from the use of isometric tasks. Among the most important of these in the context of the larger study, was the facility to define the torques necessary to displace the cursor in terms of the capability of each participant. In some arms of the study, this was set to 40% of MVT. While it is possible to manipulate the test and training forces applied during dynamic movements in a comparable fashion, the technical demands are considerable (e.g. Mackey et al. 2002; Oytam et al. 2010).

In a block of 32 trials, each of the eight targets was presented on four occasions. For a trial to commence, it was necessary that the cursor to remain in the home zone for 200 ms. The participant was instructed that following the presentation of a target, he should move the cursor to the target as quickly as possible. If the cursor was then maintained continuously within the target radius (10% of distance from origin to centre of target) for 50 ms, a tone (60 ms, 900-Hz sinusoid) signalled successful target acquisition. At this time, both the cursor and the target were extinguished. In the event that a target was not acquired within a period of 4 s following its presentation, the target was extinguished and the screen refreshed. The participant was asked to relax—to return the cursor to the home zone, immediately upon removal of the target. The next target appeared 2–3 s later.

### Pre- and post-training assessments

The right (transfer) limb was always assessed before the left (training) limb. Prior to the initial pre-training assessment for each limb, the participant undertook eight practice trials (one to each target position). In each of the subsequent assessments, each limb was employed in four blocks of 32 trials (128 trials in total for each limb). Within a block each target appeared on four occasions. There was a rest period of approximately 1 min between successive blocks. The total duration of each assessment session was about 40 min.

The pre-training assessment was conducted on a Thursday, followed by 3 days of rest. The training sessions undertaken by JG (outlined in the next section) were performed on 5 consecutive days during the next week (Monday–Friday). A post-training assessment session was conducted on the first Monday following the training week. The retention assessment took place 10 days later. A second retention assessment was undertaken for JG only after a period of 1 year (364 days) had elapsed.

The MVT values obtained during the pre-training assessment were used in both that session and (for the left limb) during the training sessions that followed. The MVT values obtained during the post-training assessment were used in both that session and in the retention assessment that took place 10 days later. The objective was that induced changes in coordination were resolved separately from any variations in torque generating capacity. A further set of MVT values were obtained and used in the second retention assessment that took place 1 year later (Table 1).

**Table 1:** The maximal voluntary torque (MVT) obtained in the pre- and post-training assessment sessions, and (for JG only) in the second retention session conducted one year following the cessation of training

JG: left limb			
Direction	Pre	Post	Retention 2
Flexion	7.11	7.15	7.94
Extension	2.91	2.85	3.14
Radial deviation	7.00	5.88	6.16
Ulnar deviation	4.80	4.89	5.45

JG: right limb			
Direction	Pre	Post	Retention 2
Flexion	9.68	8.71	7.08
Extension	4.52	4.30	5.08
Radial deviation	7.78	10.68	8.34
Ulnar deviation	6.16	5.75	4.83

Controls: left limb			
Direction	Pre	Post	
Flexion	15.59 (11.24–19.95)	13.48 (8.95–18.0)	
Extension	8.13 (5.43–10.84)	8.88 (5.45–12.31)	
Radial deviation	15.64 (10.49–20.80)	15.76 (10.11–21.42)	
Ulnar deviation	10.83(8.71–12.95)	10.78 (8.83–12.72)	

Controls: right limb			
Direction	Pre	Post	
Flexion	13.81 (10.09–17.54)	14.29 (11.30–17.28)	
Extension	10.11 (6.43–13.78)	9.87 (7.62–12.13)	
Radial deviation	16.06 (12.79–19.34)	16.15 (12.24–20.06)	
Ulnar deviation	12.46 (9.74–15.18)	12.89 (10.54–15.24)	

The values are shown separately for each limb and each direction of torque generation. The values for JG (in Nm) in each case represent the average of three trials. The values for the controls (in Nm including 95% confidence intervals) in each case represent the average of five participants (i.e. based on the individual averages of three trials)

### Training

On each of the five training days, JG performed a single session of the behavioural task using his left limb only. In each training session, JG undertook four blocks of 32 trials (128 trials in total). Within a block, each target appeared on four occasions. There was a minimum rest period of 1 min between successive blocks that JG was at liberty to extend if he desired. The total duration of each training session was approximately 20 min.

### Supplementary measurements

With a view to characterising further the abnormalities in motor control exhibited by JG, we undertook an additional set of measurements in which both the left and the right hands were instrumented with triaxial (Analog Devices ADXL335) accelerometers (secured to the base of the second metacarpals). In the course of recordings of 20 s duration (sampled at 1000 Hz), four conditions were employed (all in a seated position). In the first, JG rested his forearms and hands on his legs. In the second, both arms were held outstretched (i.e. horizontally). In the third and fourth conditions, JG flexed and extended either his left or his right elbow at a comfortable pace. In these latter conditions, five cycles of movement were performed (left limb cycle period: 4.57 s; right limb cycle period: 4.74 s).

The resultant acceleration time series were high pass filtered at 0.5 Hz to remove the component of the signal corresponding to the cyclic movements (frequency ≈ 0.2 Hz). As there are no known physiological (including pathological) frequency components of movement kinematics detectable above 40 Hz (McAuley and Marsden 2000), the time series were also low pass filtered using this frequency as a cutoff. Following linear detrending and the removal of DC bias, a Hanning window was applied to the time series. The power spectral density (PSD) function was calculated using Welch’s method, yielding an approximate frequency resolution of 0.0625 Hz (Welch 1967).

**Fig. 2:**
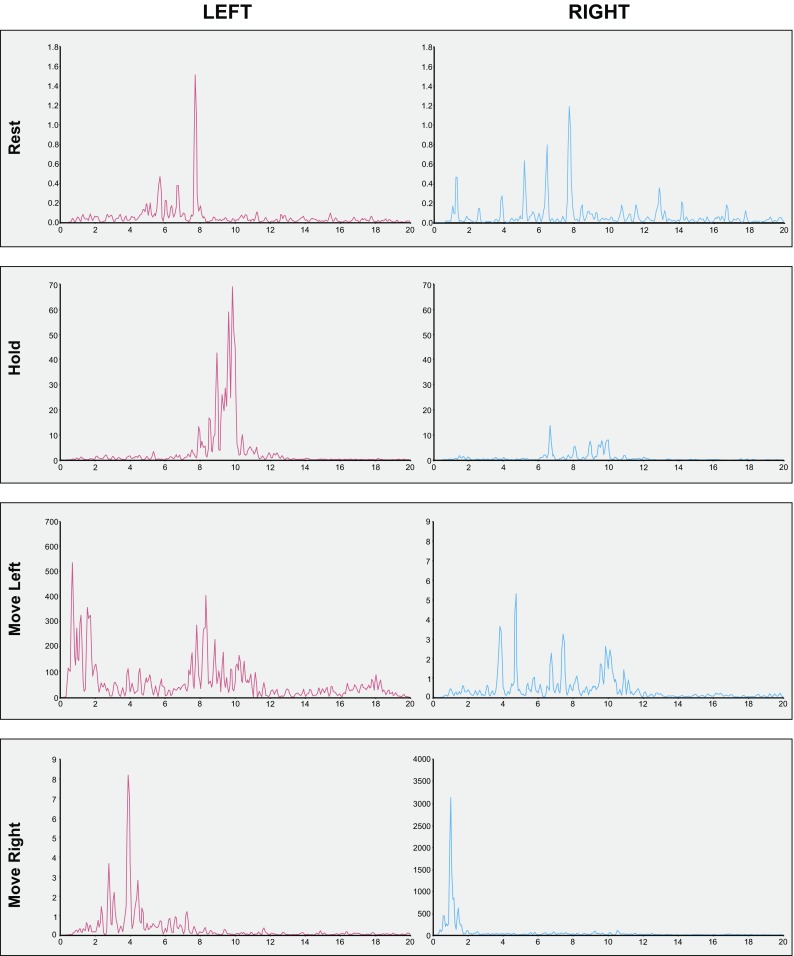
The power spectral density (PSD) functions of acceleration time series derived for participant JG from triaxial accelerometers secured to the base of the second metacarpals of each hand. In each panel, power (in arbitrary units) is represented on the ordinate and frequency (in Hz) on the abscissa. The left column depicts spectra obtained for the left limb. The right column depicts spectra obtained for the right limb. Each row corresponds to a single condition: rest; hold; move left; and move right (see text for details)

As inspection of Fig. 2 reveals, for the left limb, the frequency at which the highest power is present in the hold condition is 9.83 Hz, whereas for the right limb the highest power is present at 6.67 Hz. In contrast, in the rest condition for both limbs, the frequency for which the highest power is present is 7.75 Hz. The most obvious feature of the recording during which the right limb was moved, is the concentration of spectral power for this limb at approximately 1 Hz. In the condition in which the left limb was moved, there is by contrast a wider distribution of spectral power, including peaks in the region of 1 Hz, and between 8 and 11 Hz.

The profile thus described would tend to suggest that Parkinsonian tremor (typically in the range of 3–7 Hz) is not a distinguishing characteristic of JG’s left arm. Rather, some features of essential tremor (typically 4–12 Hz) are present. Although the differentiation of tremor types (e.g. essential versus enhanced physiological) in many classification schemes remains inexact, it is typically held that essential tremor presents clinically in both postural and kinetic task contexts. There may also be bilateral manifestation when the upper limb is engaged (e.g. Hopfner and Helmich 2017). This was a characteristic of the accelerometer recordings obtained from JG in both the hold condition, and in the condition in which the left arm was moved.

### Data analysis

For simplicity of expression in describing the data in the sections that follow, we use the term “movements” to refer to nominally isometric actions. Torque time series, defined in sensor space were low-pass filtered digitally at 6 Hz with a second-order, dual-pass Butterworth filter. Movement onset was then determined using a variant of the algorithm described by Teasdale et al. (1993), applied to a derived time series in which each sample was expressed as a displacement from the origin of the task space. Movement time (MT) was calculated as the period that elapsed from movement onset to target acquisition.

As a mean of describing the spatial characteristics of the movement trajectory, the distance between the point defined by the torque time series for each sample, and the line segment from the point defined by the torque time series at movement onset to the position of the target, was calculated. The root mean squared of all such values defined for the interval between movement onset and target acquisition was then calculated. The magnitude of this measure (r.m.s. path deviation—for which the units are percentage of MVT) indicated the degree to which the movement trajectory deviated from a straight line path between the starting position and the target position. The smoothness (continuality or non-intermittency—independent of amplitude and duration) of the trajectory for the interval between movement onset and target acquisition was defined as the spectral arc length (SAL) (Balasubramanian et al. 2012, 2015).

Heading deviation was calculated in a 10-ms window centred 100 ms following movement onset. For each sample in this interval, we calculated the angle between the following two spatial vectors. The first was the line segment from the point defined by the torque time series at movement onset to the position of the target. The second was the line segment from the point defined by the torque time series at movement onset to the point defined by the torque time series for that sample. The mean direction of the angles within the 10 ms interval was calculated using circular statistical techniques. On the basis of the assumption that modifications of the trajectory mediated by visual feedback of the cursor could not occur in advance of 100 ms following movement initiation, the heading deviation was taken to reflect the sufficiency of feedforward processes. To ensure that the heading deviation measures were restricted to trajectories that could be characterised as an initial impulse away from the starting position, we excluded instances in which the cursor velocity failed to exhibit an increasing trend (assessed using Sen’s slope) during the 100 ms interval following movement initiation. Aside from this restriction, the heading deviation was calculated for all trials. The remaining measures were derived only for trials on which the target was acquired.

### Statistical analysis

The application of traditional inferential techniques to data derived from a single case study is complicated by the fact that the errors of observations obtained from a single person tend to exhibit serial dependency. The feature (i.e. autocorrelation) violates the assumption of independence of errors that is an element of most parametric and nonparametric methods. It has the potential to generate biased descriptive and inferential statistics, and to increase the likelihood of Type I errors (Shadish et al. 2013). To address this issue, we employed a Bayesian resampling approach, whereby Markov chain Monte Carlo (MCMC) modelling was used to generate confidence (credible) intervals. The analysis was implemented in R using the MCMCglmm package (Hadfield 2010).

As the application of Bayesian resampling to circular distributions is not yet well refined, heading deviation values were analysed using semi-parametric tests designed specifically for data of this type (i.e. angles). The degree to which a set of trials (i.e. to a specific target) exhibited directionality was assessed using Rao’s Spacing Test. Contrasts between sets of trials performed prior to and following training were conducted using Rao’s Test of Homogeneity.

## Results

### Pre-training

**Fig. 3:**
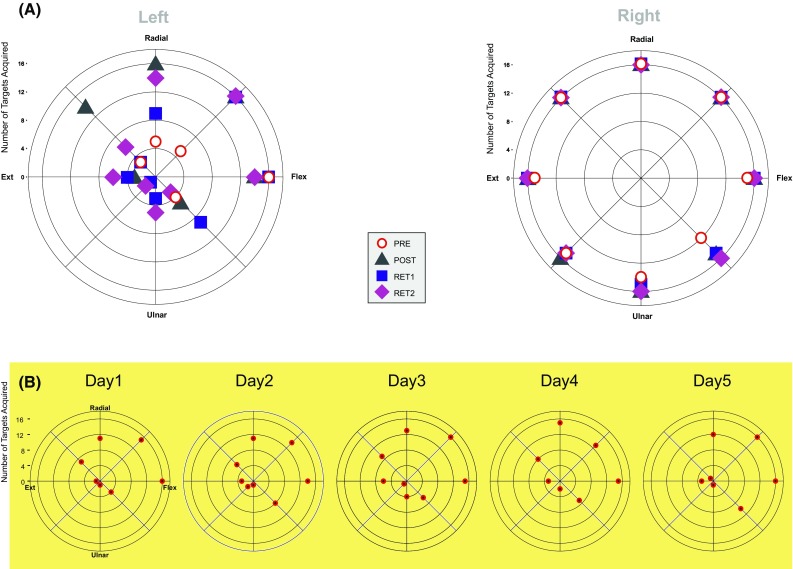
The number of instances on which a target was acquired successfully (in sixteen attempts) is shown separately for attempts made by JG using his left (**a**) and right limb (**b**), during each assessment session: Pre—red open circles; Post—green filled triangles; Retention 1—blue filled squares; and Retention 2—purple filled diamonds. In **c** the number of instances on which a target was acquired successfully (in sixteen attempts) is shown separately for each day of training undertaken by JG

The striking aspect of JG’s initial performance when using his left limb was consistent failure to produce the joint torques necessary to acquire the targets. Indeed, with respect to the three targets that required wrist extension torques, ulnar deviation torques, and their combination, JG was unable to acquire the target on any of 48 attempts (16 to each target position) (Fig. 3a—open circles). With the exception of the target that required that wrist flexion torque only be generated, the number of successful movements made to other parts of the workspace did not exceed five (from sixteen attempts).

When using his right limb, JG was able to acquire the targets on the majority (89%) of trials (Fig. 3b—open circles). The target position for which the lowest number of acquisitions was registered (12 from 16 attempts) required a combination of wrist flexion and ulnar deviation torques.

### Training

On each of the 5 days of training, JG undertook 128 trials using his left limb only. Each target was presented on 16 occasions during a training session. Although JG’s overall level of performance—in terms of acquiring targets, improved somewhat over the course of training, it remained the case that the majority of attempts were unsuccessful (Fig. 3c). This was particularly conspicuous for regions of the workspace defined by wrist extension and ulnar deviation torques. For example, JG was unable to acquire the target defined by a combination of torques in these directions on any of the 16 attempts undertaken on days 1, 4 and 5. During training sessions 2 and 3, the number of successful acquisitions was 2 and 1, respectively (Fig. 3c). With respect to the target corresponding to ulnar deviation torque only, the number of acquisitions on training days 1 to 5 was: 1, 1, 4, 2, and 1 (each from 16 attempts). A broadly similar level of performance was observed for the target that required wrist extension torque only (Fig. 3c). In short, for one quadrant of the workspace in particular, attempts during the training period to acquire targets using the left limb were characterised by persistent and pervasive failure to achieve this goal. Across the entire workspace, the overall level of successful target acquisitions did not exceed 50% on any of the 5 days of training.

### Pre- to post-training comparisons

#### Target acquisitions

*Left (training) limb*. JG’s overall level of performance during the (post) session undertaken 3 days following the cessation of training was improved (targets acquired on 52% of trials) relative to that observed prior to training (23%). It was, however, striking that the degree to which this improvement was expressed varied markedly across the workspace. When seeking during the post session to acquire targets for which wrist extension torques, ulnar deviation torques, and their combination were required, JG was able to acquire the target on only 3 of 48 attempts. (Fig. 3a—filled triangles). Although a subsequent decline in overall performance (targets acquired on 46% of trials) was observed during the retention test conducted 10 days later, 8 of 48 attempts directed to targets in the extension/ulnar deviation quadrant were successful (Fig. 3a—filled squares). In the context of a roughly equivalent (48%) overall level of performance exhibited 1 year later, 13 of 48 movements to targets in the extension/ulnar deviation quadrant were successful (Fig. 3a—filled diamonds). In summary, following 5 days of practice, JG remained unable to acquire targets that required extension torques, ulnar deviation torques, or their combination, in the majority of subsequent attempts (83%)—in spite of being afforded 4 s in which to do so.

*Right (transfer) limb*. When assessed 3 days (targets acquired on 98% of trials) following cessation of the 5 days of training undertaken by the opposite limb, 10 days (93%) and 1-year (95%) later, there were very few instances in which the task was not completed successfully (Fig. 3b).

#### Movement time

**Fig. 4:**
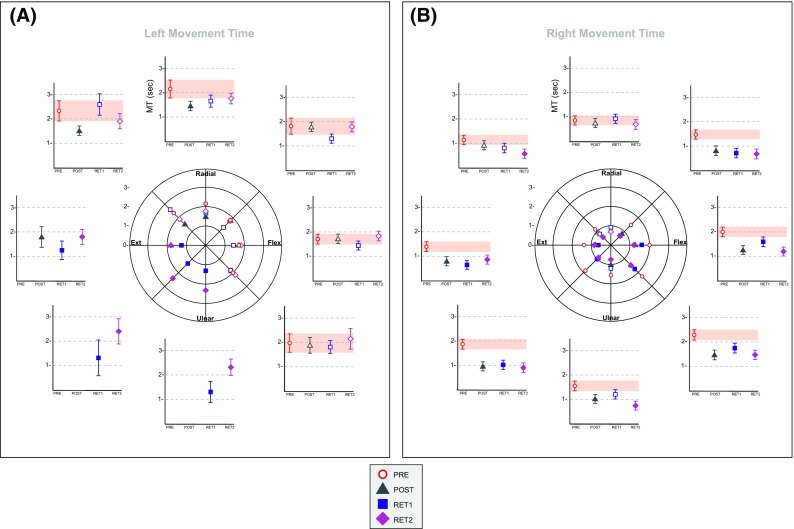
Movement Time. In the centre of each panel, the mean movement time (ms) calculated for trials on which JG acquired each target successfully, during the four separate assessment sessions: Pre—red circles; Post—green triangles; Retention 1—blue squares; and Retention 2—purple diamonds, is shown separately for the left (**a**) and right limb (**b**). The eight plots arranged around the circumference of the circle—at positions corresponding to those of the targets, indicate the means and 95% credible intervals generated by Bayesian resampling (see text for details) for each assessment session. Instances in which the credible interval for an assessment session conducted after the cessation of training, did not overlap with the credible interval obtained before training, are shown as filled symbols

*Left (training) limb*. As three of the eight target locations were not acquired by JG prior to training (i.e. in the extension/ulnar deviation quadrant), there was no basis upon which to contrast the movement times associated with the small number of successful attempts completed in the sessions that followed. With respect to two other target locations (radial deviation and radial deviation/extension), the movement times exhibited 3 days following the cessation of training (post) were shorter than those obtained during the pre session. No other changes were noted (Fig. 4a).

*Right (transfer) limb*. With the exception of targets that required radial deviation torques only for their acquisition (for which the shortest movement times were evident prior to training), the movement times obtained following the 5 days of training undertaken by the opposite limb were shorter than those exhibited prior to the commencement of training (Fig. 4b).

#### Path deviation

*Left (training) limb*. There was only one instance of a difference between the value recorded prior to training [lower CI = 0.02; upper CI = 0.12 (units %MVT)] and that obtained following training. A larger value was observed during the retention1 session for the radial deviation/extension target (= 0.19%MVT).

*Right (transfer) limb*. With respect to targets that required a combination of wrist flexion and radial deviation torques for their acquisition, the path deviation values were markedly lower following training (post = 0.09; retention1 = 0.09; retention2 = 0.09%MVT) than prior to training (lower CI = 0.15; upper CI = 0.19). With the exception of the retention2 session (conducted 1 year later), this was also the case for the extension target (prior to training: lower CI = 0.11 upper CI = 0.16; post = 0.08; retention1 = 0.08).

#### Spectral arc length (SAL)

*Left (training) limb*. There were no instances following training in which SAL values fell outside of the 95% credible intervals derived on the basis of the pre-test values.

*Right (transfer) limb*. With respect to targets that required a combination of wrist extension and ulnar deviation torques for their acquisition, SAL values (prior to training: lower CI = − 10.97; upper CI = − 9.06) were of lower magnitude (post = − 6.31; retention1 = − 7.03; retention2 = − 5.84) following training of the opposite limb—indicating that the trajectories were smoother. This was also the case for movements directed to flexion targets (prior to training: lower CI = − 9.00; upper CI = − 7.13; post = − 5.01; retention1 = − 6.82; retention2 = − 5.45), and to a much lesser degree for the combinations of wrist flexion and radial deviation.

#### Heading deviation

**Fig. 5:**
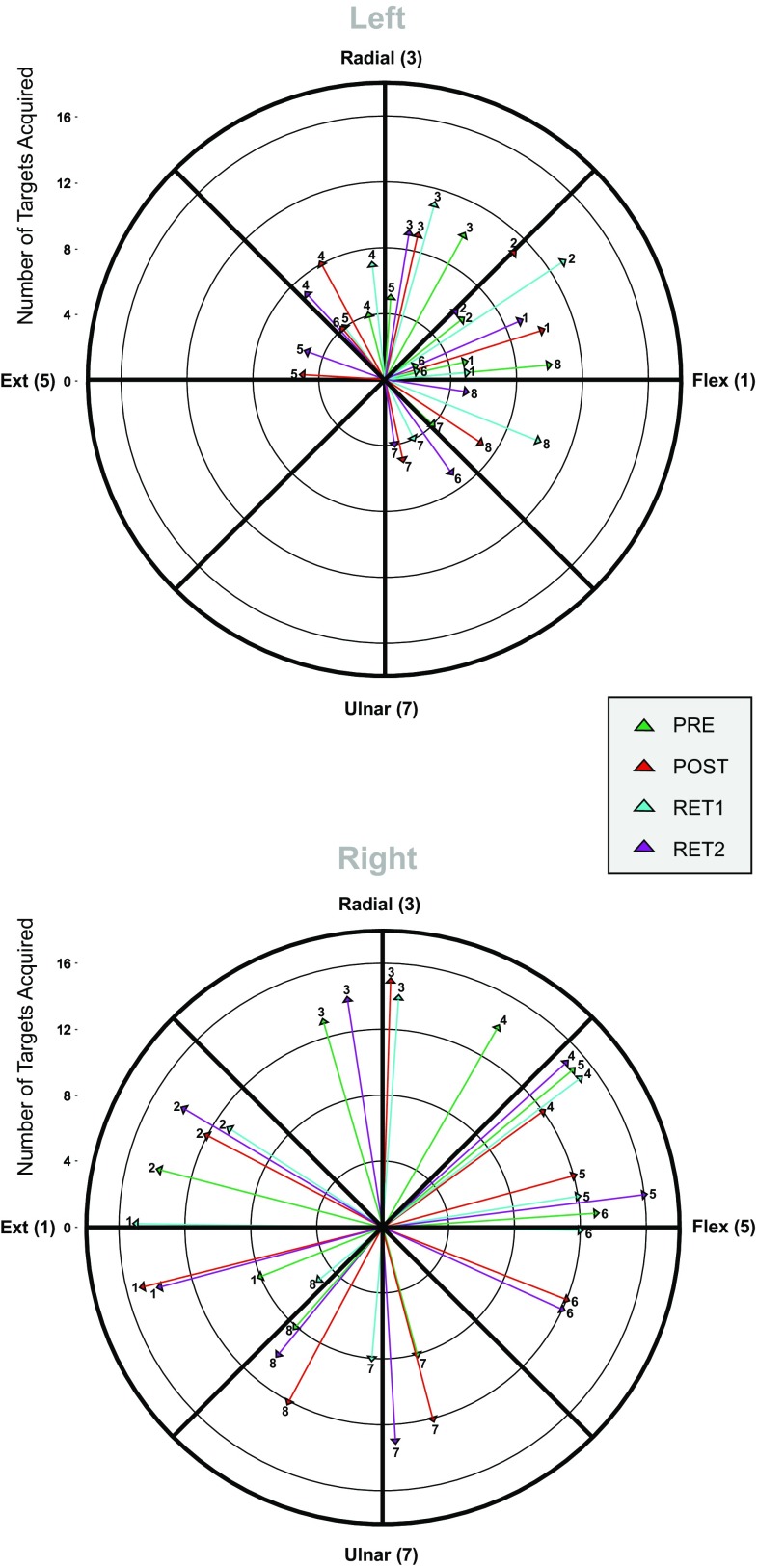
Heading deviation. This measure expresses the difference in angle between the vector defined by the torque impulse in a 10-ms window centred at 100 ms following movement onset, and a straight line segment from the point defined by the torque time series at movement onset to the position of the target. The length of each vector represents the number of trials (maximum = 16) on which the cursor velocity (i.e. rate of torque development) showed an increasing trend during the 100 ms interval following movement onset. Each vector is labeled according to the corresponding target. The numeric equivalents of the four single degree of freedom targets (in the anatomical coordinate system) are denoted on the circumference of the circle. The angle subtended by the arc between each vector and its corresponding target radius (i.e. sharing the same numeric code), from the origin, indicates the deviation of the initial heading (of the torque impulse) from a direct line between the starting position and the target. Note that for the left limb (**a**) Flexion corresponds to target position 1, and Extension to target position 5. For the right limb (**b**), Extension corresponds to target position1, and Flexion to target position 5. Red vectors represent the heading deviations obtained during the pre session; green vectors those obtained during the post session; blue vectors the Retention 1 session; and purple vectors the Retention 2 session. In some cases (i.e. for the left limb—see Table 2), the vector may not correspond to a statistically reliable measure of central tendency. A decrease in the subtended angle from pre (red vector) to post/retention (green, blue, purple vectors) indicates that the initial torque impulse aligned more closely with the direct path between the starting position and the target

*Left (training) limb*. As inspection of Fig. 5a suggests, when using his left limb, JG was largely incapable of generating torques directed towards targets located in the wrist extension/ulnar deviation quadrant. Indeed, the variability of the headings generated during attempts to acquire the wrist extension and extension/ulnar deviation targets during the pre- and post-training sessions, was sufficient to preclude the reliable estimation of a “mean” direction (Table 2). This was also the case during the retention session (not tabulated). A reduction in the magnitude of the heading deviation following training was observed for targets requiring only radial deviation, and the combination of flexion and ulnar deviation.

**Table 2:** The central tendency (mu) and lower and upper 95% confidence limits (Cl) of the heading deviation measures (in degrees) obtained for the pre- and post-training assessment sessions

Left Limb							
	Pre lower CI	Pre mu	Pre upper CI	Post lower CI	Post mu	Post upper CI	Pre versus post
Flx	8.4	13.9	19.0	11.1	19.0	26.2	Homogenous
Flx/Rad	− 13.2	− 4.8	4.9	− 6.4	1.4	9.4	Homogenous
Rad	− 35.7	− 26.7	− 17.7	− 22.3	− 10.8	− 0.8	Non-homogeneous
Ext/Rad	− 79.9	− **29.1**	21.1	− 41.5	− 14.2	4.1	Homogenous
Ext	− 150.6	− **92.1**	− 3.0	− 24.2	− **1.0**	54.6	Homogenous
Ext/Uln	76.9	**159.0**	241.0	− 153.8	− **93.2**	− 32.8	Homogenous
Uln	26.8	48.8	67.8	9.5	14.5	19.5	Homogenous
Flx/Uln	35.7	51.6	64.5	− 1.5	13.4	26.7	Non-homogeneous

Right limb							
	Pre lower CI	Pre mu	Pre upper CI	Post lower CI	Post mu	Post upper CI	Pre versus post
Flx	48.4	39.8	30.4	20.1	15.1	10.1	Non-homogeneous
Flx/Rad	23.1	15.1	1.3	0.4	− 9.0	− 18.7	Non-homogeneous
Rad	23.9	16.0	9.1	5.1	− 1.9	− 9.1	Non-homogeneous
Ext/Rad	39.3	30.4	21.6	24.1	17.3	10.8	Non-homogeneous
Ext	33.5	22.1	12.4	20.8	13.8	5.8	Homogenous
Ext/Uln	13.2	3.7	− 5.9	22.8	16.6	10.9	Non-homogeneous
Uln	25.2	15.0	6.9	17.9	14.7	11.6	Homogenous
Flx/Uln	61.6	48.7	36.5	31.9	23.7	16.2	Non-homogeneous

The values are shown separately for each limb and target position

Values in bold indicate instances in which Rao’s Spacing Test indicated the absence of directionality. The Pre versus Post contrasts were conducted using Rao’s Test of Homogeneity. The designation “homogenous” indicates that the pre- and post-training deviation measures could not be discriminated reliably. With respect to Fig. 5, negative values indicate that the heading is displaced clockwise relative to the target

*Right (transfer) limb*. With respect to all torque impulses generated during the pre-training assessment session, the heading deviations were characterised by a “clockwise” bias (Fig. 5b). Expressed in anatomical terms: impulses directed to flexion targets were biased towards radial deviation; impulses directed to extension targets were biased towards ulnar deviation; radial deviation towards extension; and ulnar deviation towards flexion. With the exception of impulses directed towards combined wrist extension/ulnar deviation targets, the magnitude of this bias was smaller during the post-training assessment (Table 2). In other words, the initial heading was more closely aligned with the direction of the target.

### Control group comparison

It is reasonable to seek an indication that the changes in the performance of the right limb exhibited by our case study participant, were attributable to the training undertaken by the left limb—rather than to the “practice” effects associated with the (pre, post, retention) testing regime. A comparison was therefore made with five right-handed older (aged 85, 67, 72, 79 and 81) male “control” participants drawn from the main trial (i.e. those most closely matched), who also performed the pre, post, and retention assessments—by applying torques at 20% MVC (i.e. identical to those undertaken by JG). In the course of their five “training” sessions, these participants were required to simply count (“in their head”) the number of occasions upon which an infrequent (~ 15–25% probability) blue target appeared at random among standard green targets over the course of each set of 128 trials, and report this value at the end of the set. Their limbs were placed in the manipulanda in the usual fashion, however no movements were generated—the participants sat passively, and the cursor that would otherwise indicate wrist joint torques was not displayed.

For the present purposes, attention is restricted to changes in MT (Table 3) from pre to post, and from pre to retention, respectively (expressed as a percentage of the corresponding pre-training values). As a basis upon which to draw inferences, the mean and 95% confidence intervals for the values obtained for the five control participants (i.e. separately for each target) were calculated. This allowed the following question to be posed: did the percentage change in MT exhibited by JG for a given target lie within the confidence interval defined for the other five participants?

**Table 3:** The means of the times required to acquire the target (“movement time” in seconds), obtained for JG and for the five control participants—when the right limb was used

JG	Pre	Post	Retention	Retention 2
e013 (85 years)	1.52	0.96 (− 36.6%)	1.02 (− 32.5%)	0.87 (− 43.1%)

Controls	Pre	Post	Retention	
e013 (85 years)	1.01	0.88 (− 12.8%)	0.99 (− 2.2%)	
e019 (67 years)	0.82	0.75 (− 8.5%)	0.61 (− 25.1%)	
e038 (72 years)	0.59	0.64 (+ 8.6%)	0.48 (− 18.5%)	
e062 (79 years)	0.62	0.55 (− 10.7%)	0.64 (+ 3.8%)	
e073 (81 years)	0.90	0.94 (+ 4.2%)	0.84 (− 7.0%)	

The values were calculated separately for individual participants by averaging the respective median values obtained from the sixteen trials directed to each of the eight targets. The values shown in brackets correspond to the percentage change in time expressed relative to the value obtained at the start of testing (“Pre”)

**Fig. 6:**
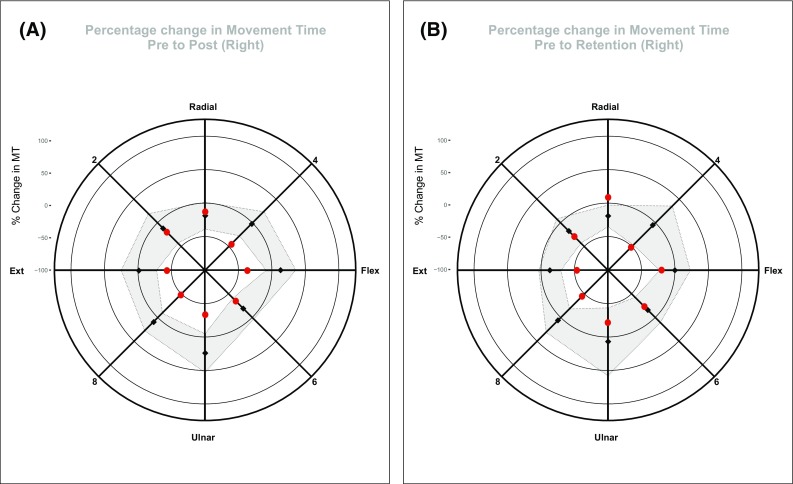
Control group comparison. The change in mean movement time (ms) from pre-training to post-training (**a**), and from pre-training to the first retention session (**b**) (expressed as a percentage of the corresponding pre-training values), exhibited by JG for each of the eight target positions is shown as red filled circles. The corresponding means obtained for a group of five age- and gender-matched control participants—who did not undertake training of the left limb, are shown as black filled diamonds. The grey-shaded regions represent the 95% confidence intervals calculated across the five control participants (i.e. separately for each target)

When the change in MT assessed at post is considered (Fig. 6a), it becomes apparent that for five of the eight target locations, the gains achieved by JG were discernably greater than those exhibited by the control group (i.e. the values obtained for JG were outside of the 95% confidence bands defined around the control group means). With respect to the targets that required radial deviation only, and combined extension and radial deviation torque—for which JG did not exhibit an advantage, inspection of Fig. 4b reveals the presence of a floor effect. During the pre-training session, the MTs for these targets were lower (in most cases distinctly so) than for all other targets. It seems likely therefore that in acquiring targets that required radial deviation only, and combined extension and radial deviation torques, there was simply little scope for JG to further improve his performance, i.e. via interlimb transfer arising from the training performed by the opposite limb. It is particularly notable that the greatest gains in right limb performance exhibited by JG (relative to the control group) were for those targets that he could not acquire consistently when using his left limb during the course of training.

A broadly similar pattern of outcomes was apparent when the changes in performance from pre-training to retention were considered (Fig. 6b). For three of the eight target positions, the gains exhibited by JG were greater than those of the control group. The somewhat lower reliability in the expression of these differences is attributable to the larger confidence intervals obtained for the control group at retention. It might also be noted that confidence intervals generated on the basis of five participants will necessarily be relatively broad.

## Discussion

In the present case study, we sought to examine the following question: does interlimb transfer occur if and only if the training movements are executed? The opportunity to do so came about serendipitously as a consequence of the profound difficulty exhibited by JG in generating wrist flexion–extension and ulnar deviation torques as the means to acquire visual targets. To put the extent of this deficit in context, over the course of 48 attempts during the pre-test session, and 240 attempts during the training sessions, JG was able to acquire the three targets that required ulnar deviation torques wrist extension torques, and their combination, on approximately one in nine attempts. When assessed again 3 days following the end of the training period, JG was unable to acquire either the ulnar deviation target or the target that required combined ulnar deviation and extension torques—in the course of 32 attempts. His rate of success in acquiring extension targets was marginally greater (3 of 16 attempts).

The lack of success was not simply due to a deficit in generating joint torque per se. The magnitude of the torque required to acquire each target was scaled to 20% of JG’s assessed capability. Indeed, in the MVT task, JG was able to separately generate extension and ulnar deviation torques. Although it was of lower magnitude (≈ 65%) than that produced by his right limb, the value recorded for wrist extension was within 2.5 standard deviations of the (left side) mean for European males in the range 60 to 69 years (Decostre et al. 2015). The ulnar deviation MVT for his left limb was 78% and 85% of values recorded for the right limb during the pre and post sessions, respectively. Rather, JG’s exceptional functional deficiencies were expressed when precise and coordinated wrist extension and ulnar deviation torques were required.

In spite of the fact that JG was mainly incapable of executing movements in an entire quadrant of the workspace during the 5 days of training undertaken by the left limb, when the performance of the untrained right limb was assessed subsequently, dramatic improvements in performance were evident across the workspace. Most notably, decreases in the time required for the right limb to acquire targets in the wrist extension/ulnar deviation quadrant were particularly pronounced. In the case of the target that required combined wrist extension and ulnar deviation torques, the magnitude of the change in movement time exceeded that of all the other target locations. The extent of this specific gain in performance (i.e. to this target) also exceeded most conspicuously the variations in movement time exhibited by a control group that did not train (Fig. 6a).

It is possible to conceive of the possibility that there were gains in right limb performance accrued by JG during the pre-training assessment, that were somehow reactivated/consolidated (e.g. Amar-Halpert et al. 2017) by the left limb training sessions, and that this accounted for a level of performance that was superior to that of the controls. The extremely meagre overall changes in movement time (a mean of − 1% across all targets) exhibited by the control group suggest, however, that the pre-test assessment procedures did not give rise to a significant degree of learning. In this context, the 48% decrease (from pre- to post-training) in movement time achieved by JG, when using his right limb to acquire a target (combined wrist extension and ulnar deviation) that he was entirely incapable of capturing with his left limb (i.e. during the post-test assessment) is particularly striking. The gains achieved for the right limb in acquiring the extension only (− 43%) and ulnar deviation only (− 34%) targets are similarly impressive.

Another question that arises is whether the interlimb transfer was attributable to actions that were directed unsuccessfully to targets in specific spatial locations, or rather reflects the generalisation of gains achieved through successful training movements that were performed in other parts of the workspace. Perhaps the most obvious point that can first be made in relation to generalisation of motor learning, is that the gains realised for the untrained variant of a task seldom approach the magnitude of benefits derived for the trained variant (de Rugy 2010). In the present case, the largest relative gain in performance for the untrained limb was for combined wrist extension and ulnar deviation torques (48% reduction in movement time). This represents 162% of the mean change in performance exhibited for targets located in the other quadrants. The corresponding values for the wrist extension and ulnar deviation targets were 144% and 114%, respectively. While the extent of these gains in performance does not preclude the possibility that there was generalisation of the benefits derived from training movements performed successfully by the opposite limb in other parts of the workspace, it does suggest that other factors were also involved.

Reductions in the time taken for the right limb to acquire targets were generally accompanied by decreases in the deviation of the initial torque impulse from a straight line trajectory to the target (Table 2). The exception in this regard was the target that required combined wrist extension and ulnar deviation torques for its acquisition. For this target however, the torque trajectories generated by JG were considerably smoother (SAL measure) following the training performed by the opposite limb. In other words, constraints on the nature of the adaptations that occur in the context of interlimb transfer are not necessarily imposed equivalently across the workspace.

Although to the best of our knowledge JG has not sought or received a formal neurological diagnosis, the presentation of ataxic symptoms (including abnormalities of gait, difficulties with speech, and with dressing) in association with characteristics of essential tremor, may suggest some degree of disruption of cerebellar function. Nonetheless, neural deficits of other origin (such as those associated with Parkinson’s disease) or musculoskeletal pathology cannot be excluded. Beyond their unilateral (left) manifestation, the most striking aspect of the difficulties in motor control exhibited by JG, was the degree to which their expression was contingent upon the specific patterns of muscle engagement that were required. The generation of rapid and precise wrist extension and ulnar deviation torques posed an almost insurmountable challenge. In contrast, the production of wrist flexion torques, or combinations of wrist extension and radial deviation torques was accomplished more readily.

The circumscribed nature of the deficits in generating coordinated goal directed joint torques raises the possibility that the interlimb transfer effects—which were evident both for targets within the quadrant for which JG experienced difficulties, and for targets in the other quadrants, were contingent upon training-related refinements of motor planning (rather than of execution per se). The adequacy of motor planning is often assessed on the basis of the initial directional error (e.g. Kagerer et al. 1997) or angular error (e.g. Hinder et al. 2008a) during the first 80–100 ms of the response. It is generally assumed that these “errors” (i.e. relative to a straight line path to the target) are indicative of the accuracy of the feedforward commands (e.g. Berger and d’Avella 2014). As inspection of Fig. 5a reveals, torque impulses generated by the left limb with the intent of acquiring targets in the wrist extension/ulnar deviation quadrant were not in the appropriate direction. Indeed, for the extension and combined extension/ulnar deviation targets, the variation of the heading direction across trials was sufficiently large as to preclude reliable estimates of a “mean” (Table 2). With respect to all targets in this quadrant, the pre- and post-training heading deviation measures could not be discriminated reliably. In other words, in terms of this measure, there was no apparent expression of learning.

For the untrained limb, the largest changes in overall level of performance (i.e. in movement time) were exhibited for impulses directed to targets in the wrist extension/ulnar deviation quadrant. They were not, however, accompanied by decreases in the heading deviation registered at 100 ms. Indeed, for targets that required combined wrist extension and ulnar deviation, the heading deviation was greater following training of the left limb. Evidently therefore, training-induced alterations in the adequacy of motor planning (at least to the extent that these are captured by the direction of the initial impulse) were not the basis of the interlimb transfer of functional capacity exhibited by JG.

When he attempted to acquire targets in the extension/ulnar deviation quadrant, feedback conveying the terminal accuracy of his attempts was seldom available to JG. During the period of 4 s that was permitted for each attempt, he did, however, receive continuous visual feedback of the cursor (representing the torque being applied) relative to the target. Within limits imposed by his deficiencies in coordination, visual feedback of target proximity may have facilitated a form of latent learning that was exploited when movements of the opposite limb were performed subsequently.

There is an extensive body of work that concerns the distinction between ‘learning’ and ‘performance’ (Soderstrom and Bjork 2015). This literature makes clear that learning can occur even when there are no discernible changes in performance. Numerous factors can mask the occurrence of such latent learning, including a lack of reinforcement, fatigue, and the imposition of complex demands that increase the frequency of errors (Soderstrom and Bjork 2015). It might be argued that JG did not exhibit latent learning in the strictest sense as for two target positions a post-training improvement in the capability of his left limb was registered (Fig. 4a). It was, however, the profound increases in the task-relevant functional capacity of his untrained right limb i.e. the interlimb transfer that provided compelling evidence that latent learning had occurred. Evidently the full extent of this learning could be registered only when it was expressed via an unimpaired effector system. Typically, latent learning is revealed when parameters of the task are altered relative to those of the initial training environment, in such a manner that performance is enhanced (e.g. reinforcement is introduced). In our study, the alteration necessary to reveal learning was not of a parameter such as effort, attention, task complexity, or arousal, but rather the use of a motor system—the opposite limb, that allowed for successful task execution. It can be concluded that latent learning—as it is expressed via interlimb transfer, occurs even when training movements are not executed. For JG, it was his sustained attempts to acquire the targets, rather than his level of success in these endeavours, that appears to be have been the basis for the remarkable learning that he displayed.

The quality of learning that occurred over the course of the 5 days of training is further emphasised by its longevity. When reexamined 1 year later, JG for the most part acquired targets (with his right limb) at least as rapidly as he had done when assessed 3 days following the cessation of training (Fig. 4b). In all cases, a reliable decrease in movement time from pre- to post-training was accompanied by a similar robust difference being registered the following year. Indeed, in one instance (for combined extension/radial deviation targets), the movement times recorded one year later were also markedly lower than those obtained in the post-training session.

It should be emphasised that this study has several limitations. For example, three of the participants included in the control group were older by JG by more 10 years. With the obvious exceptions of age cohort (young or older) and gender, the researchers undertaking the assessments were blinded as to the group allocation of individual participants during the conduct of the study. As it was only upon the conclusion of the study that we were in a position to match control participants to the characteristics of JG (i.e. older male; pre and post assessments at 20% MVT), there was no scope to ensure greater similarity in terms of age. Although the size of the control group precludes a quantitative analysis of age-related variation, inspection of Table 3 fails to suggest that the oldest individuals (≥ 79 years) exhibited changes in performance that were distinct from those of the group closest in age to JG.

It is a further limitation that a clinical assessment of JG was not available. The observations that were made, and the measurements that were obtained, do not provide a sufficient basis upon which to resolve the aetiology of his condition. As noted previously, JG did not express a desire for the research team to make representations that may have led to specialist medical consultations or assessment. As a consequence, it remains undetermined whether his movements were mediated by a (relatively) normal nervous system acting on an impaired musculoskeletal system, or by a compromised nervous system acting on a relatively normal musculoskeletal system. Although the balance of evidence would appear to favour the latter interpretation, without further diagnostics tests, it is not possible to distinguish causes.

One might also pose the question: are isometric tasks distinct from free motion tasks, in terms of the nature the learning to which they give rise? While there was minimal joint rotation, the muscles involved in the isometric task necessarily shortened and lengthened. Cutaneous and tendon organ receptors are likely to have provided additional correlated proprioceptive feedback. It is acknowledged that the mapping between the joint torques and cursor movements was arbitrary, and it was a requirement that this was learned. Necessarily, however, this is the case whenever one learns to use many new tools (e.g. a computer mouse). It is assumed, therefore, that the task shared the key features of most voluntary movements—a set of neural commands generated with the intent of evoking a particular outcome (with respect to which visual and proprioceptive sensory feedback is available). There is also empirical evidence to support the contention that isometric tasks—such as the one employed in the present study, give rise to forms of learning that appear equivalent to those which characterise dynamic tasks (e.g. Hinder et al. 2008b, 2010; Rotella et al. 2013; Shemmell et al. 2005a, b), and which in some cases transfer directly to free motion (Baek and Okamura 2015; Melendez-Calderon et al. 2017). It is nonetheless a premise of the present study that an isometric task can be used, i.e. without loss of generality, to address the question that was defined.

The presence of these potential limitations does not mitigate the key findings of the present study. When JG used his impaired limb, marked deficiencies in movement execution were prominent both throughout training, and when his performance was assessed following the cessation of training. In spite of his frequent and persistent failure to accomplish the training task, a significant degree of learning was nonetheless manifested—i.e. when the performance of his unimpaired (untrained) limb was assessed. Evidently, it is not necessary for training movements to be executed in order for interlimb transfer to occur. While the changes in brain connectivity and local neuronal adaptations that mediate the long-term retention of functional capabilities acquired initially via interlimb transfer remain to be resolved (cf. Ruddy et al. 2017), it appears that in JG these were intact, and that they were dissociated from the deficits that gave rise to his difficulties in motor control.
